# Risk of Advanced Colorectal Neoplasia According to Age and Gender

**DOI:** 10.1371/journal.pone.0020076

**Published:** 2011-05-24

**Authors:** Frank T. Kolligs, Alexander Crispin, Axel Munte, Andreas Wagner, Ulrich Mansmann, Burkhard Göke

**Affiliations:** 1 Department of Medicine II, University of Munich, Munich, Germany; 2 Institute of Medical Informatics, Biometry, and Epidemiology, University of Munich, Munich, Germany; 3 Bavarian Association of Compulsory Health Insurance Physicians, Munich, Germany; Technische Universität München, Germany

## Abstract

**Background:**

Colorectal cancer (CRC) is one of the leading causes of cancer related morbidity and death. Despite the fact that the mean age at diagnosis of CRC is lower in men, screening by colonoscopy or fecal occult blood test (FOBT) is initiated at same age in both genders. The prevalence of the common CRC precursor lesion, advanced adenoma, is well documented only in the screening population. The purpose of this study was to assess the risk of advanced adenoma at ages below screening age.

**Methods and Findings:**

We analyzed data from a census of 625,918 outpatient colonoscopies performed in adults in Bavaria between 2006 and 2008. A logistic regression model to determine gender- and age-specific risk of advanced neoplasia was developed. Advanced neoplasia was found in 16,740 women (4.6%) and 22,684 men (8.6%). Male sex was associated with an overall increased risk of advanced neoplasia (odds ratio 1.95; 95% confidence interval, CI, 1.91 to 2.00). At any age and in any indication group, more colonoscopies were needed in women than in men to detect advanced adenoma or cancer. At age 75 14.8 (95% CI, 14.4–15.2) screening, 18.2 (95% CI, 17.7–18.7) diagnostic, and 7.9 (95% CI, 7.6–8.2) colonoscopies to follow up on a positive FOBT (FOBT colonoscopies) were needed to find advanced adenoma in women. At age 50 39.0 (95% CI, 38.0–40.0) diagnostic, and 16.3 (95% CI, 15.7–16.9) FOBT colonoscopies were needed. Comparable numbers were reached 20 and 10 years earlier in men than in women, respectively.

**Conclusions:**

At any age and independent of the indication for colonoscopy, men are at higher risk of having advanced neoplasia diagnosed upon colonoscopy than women. This suggests that starting screening earlier in life in men than in women might result in a relevant increase in the detection of asymptomatic preneoplastic and neoplastic colonic lesions.

## Introduction

Colorectal cancer is among the leading causes of cancer related morbidity and mortality in the United States and Europe [1]–[3]. Screening for colorectal cancer by fecal occult blood testing (FOBT), flexible sigmoidoscopy, and colonoscopy can detect precancerous lesions and cancers at early stages [4]–[6]. However, colonoscopy is the most sensitive test available and studies have demonstrated substantial reductions in the expected risk of colorectal cancer during long-term follow-up after screening colonoscopy [6]–[8].

For the average risk population guidelines usually recommend starting screening at the age of 50 years [9], [10]. There are separate recommendations for individuals at increased risk because of a family history of colorectal cancer, a genetic predisposition such as familial adenomatous polyposis coli (FAP) and hereditary nonpolyposis colorectal cancer (HNPCC), a personal history of inflammatory bowel disease or prior neoplasia. Recently, the American College of Gastroenterology has acknowledged the increased risk of male African Americans [11] and recommends starting screening at an age of 45 years in this group [12]. Although lifetime risk of colorectal cancer appears to be similar among men and women, the age-adjusted rate of advanced adenoma that is detected during screening is higher in men and colorectal cancer occurs at an earlier age in men than in women [2], [13]–[18].

Despite these facts it is uncertain whether colorectal cancer screening should start at an earlier age in men than in women. Our current knowledge on gender as a risk factor for colorectal cancer is based on screening populations, high-risk populations, cancer registry data, and mortality statistics [16], [18], [19]. Still, we have limited information on the frequency of advanced adenoma, the common precursor lesion and main target lesion of colorectal cancer screening, in the general risk population under the age of 50 years [20]. To provide data on the age- and gender-specific risk of advanced adenoma, we analyzed population-wide data from a census of outpatient colonoscopies performed in Bavaria in the years 2006 through 2008. We determined the age-specific risk of advanced adenoma and cancer in men and women and developed logistic regression models to predict the number of colonoscopies needed to detect advanced adenoma and cancer, i.e. the number of persons who have to undergo colonoscopy to detect one lesion, depending on age and gender.

## Methods

### Study Design

This study is based on a census of outpatient colonoscopies obtained as part of routine care in Bavaria, Germany, between January 2006 and December 2008. The complete documentation of out-patient colonoscopies is required for reimbursement. No individual-related data are contained in the data base. Contents and the procedure of data collection are regulated by the “Krebsfrüherkennungs-Richtlinie” and have been approved by the governmental authority for data protection of Bavaria.

More than 83% of the Bavarian population of 12.5 million are members of sickness funds forming the compulsory health insurance (CHI) system [21], [22]. Reimbursement for outpatient care of CHI patients requires affiliation of a physician with the Association of CHI Physicians. Since January 2006 all outpatient colonoscopies of CHI patients and their histopathological results are documented electronically in a central database via the internet. Photographic documentation of the cecal landmarks is required. A similar federal database covers only screening colonoscopies, while, uniquely, the Bavarian database documents all colonoscopies. Only completely documented colonoscopies are reimbursed. Since October 2002, all CHI insurants aged 55 years or older are entitled to two screening colonoscopies at an interval of 10 years. In Bavaria, the cumulative participation rate of CHI insurants in screening colonoscopy from 2003 through 2008 was 15.1% for women and 13.8% for men, respectively. In the years 2006 through 2008 a total number of 1,955,790 FOBT tests were reimbursed in Bavaria. In 2008, 13.6% of male and 21.9% of female CHI insurants between age 50 and 74 received an FOBT.

From January 2006 through December 2008, a total of 802,966 colonoscopies were documented in the database. We excluded 1,997 colonoscopies in patients aged <18 years and 28,435 examinations of individuals aged ≥80 years. Additional 107,204 examinations were excluded because they were done for post-adenoma or post-carcinoma surveillance. After excluding 2,906 doublets and 36,506 records of repeat examinations, a total of 625,918 colonoscopies were included in the analysis. In case of repeat examinations, the index colonoscopy was included in the analysis. Biopsy specimens were evaluated by local pathologists using common criteria. The findings on colonoscopy were categorized on the basis of the most advanced lesion found. Advanced adenoma was defined as adenoma that was at least 10 mm in diameter, had high-grade dysplasia, had villous or tubulovillous histologic characteristics, or any combination thereof [23]. Advanced neoplasia was defined as cancer or advanced adenoma.

### Statistical Analysis

**Figure 1:**
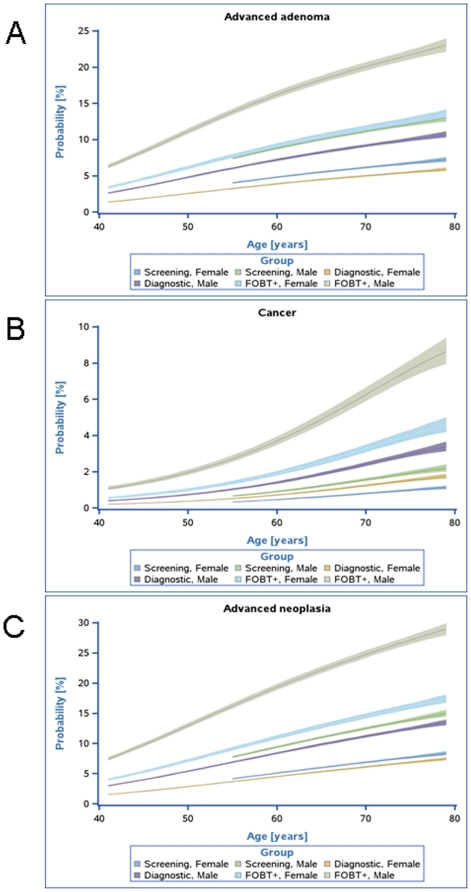
Predicted probabilities of advanced adenoma (A), cancer (B), and advanced neoplasia (C) in screening and diagnostic groups according to age and gender. The bands mark the corresponding 95% confidence intervals.

Data were described using appropriate measures of location and dispersion. Statistical inferences on the risks of advanced adenoma, cancer, and advanced neoplasia were based on logistic regression models including a fixed set of independent variables: Gender, age, age squared, age cubed (to account for the obvious nonlinear relations), indication, completeness of the examination, and intravenous sedation. Intravenous sedation was included because sedation has previously been shown to be associated with completeness of colonoscopy [24]. More detailed information is provided with the supplementary methods S1. While the presentation of the number of colonoscopies needed to identify cancer or advanced adenoma and figure 1 were restricted to the relevant age group of potential screening clients (40 to 79 years of age), all patients were included in the analyses. We did not use a data-driven variable selection algorithm. Model fit was assessed graphically by plotting the observed frequencies and the probabilities predicted from the regression equations. Following Regula and co-workers [16], the number of colonoscopies needed to detect one lesion was defined as the reciprocal of the probability predicted by the logistic regression model. Analogously, 95% confidence limits were calculated as reciprocals of the 95% confidence limits for the predicted probabilities. All analyses were performed using SAS version 9.2 for Linux (SAS Institute Inc., Cary, NC, USA).

## Results

A total of 625,918 colonoscopies performed in adults between 18 and 79 years of age were included in the analysis. 407,364 colonoscopies were done for diagnostic reasons, 180,904 were primary screening colonoscopies performed between 55 and 79 years of age, and 37,650 colonoscopies were screening colonoscopies performed to follow up on individuals positively pre-screened by FOBT (FOBT+ colonoscopies). 363,200 participants were female and 262,718 were male. The characteristics of the study population are listed in Table 1. Indications for diagnostic colonoscopies are listed in Table S1. 95% of the diagnostic colonoscopies, 93.7% of FOBT+ colonoscopies and 92.8% of the screening colonoscopies were performed with intravenous sedation. In all groups, women more frequently received intravenous sedation. The cecum was reached in 97.3% of diagnostic, 97.5% of FOBT+, and 98.3% of screening colonoscopies. Acute complications were rare, with bleedings occurring overall in 0.16%, perforations in 0.02%, and cardiorespiratory complications in 0.03% of colonoscopies, respectively.

**Table 1:** Demographic characteristics for the 625,918 participants aged 18 to 79 years in diagnostic and screening colonoscopy.

Characteristic	Screening Colonoscopies		FOBT-pos. Colonoscopies		Diagnostic Colonoscopies	
	Men(N = 81,078)	Women(N = 99,826)	Men(N = 16,979)	Women(N = 20.671)	Men(N = 164,661)	Women(N = 242,703)
Age – yr						
Range	55–79	55–79	18–79	18–79	18–79	18–79
Mean ± SD	64.3±6.3	63.8±6.4	58.8±11.9	58.3±12.0	52.1±14.4	52.1±14.9
Age group – no. (%)						
18–39	-	-	967 (5.7)	1,324 (6.4)	32,164 (19.5)	49,117 (20.2)
40–44	-	-	1,004 (5.9)	1,272 (6.2)	18,478 (11.2)	25,262 (10.4)
45–49	-	-	1,889 (11.1)	1,822 (8.8)	21,078 (12.8)	29,081 (12.0)
50–54	-	-	2,533 (14.9)	3,634 (17.6)	22,879 (13.9)	32,650 (13.5)
55–59	23,785 (29.3)	32,526 (32.6)	2,033 (12.0)	2,588 (12.5)	15,517 (9.4)	23,784 (9.8)
60–64	18,574 (22.9)	22,537 (22.6)	2,029 (12.0)	2,457 (11.9)	14,024 (8.5)	21,305 (8.8)
65–69	20,862 (25.7)	24,430 (24.5)	2,941 (17.3)	3,438 (16.6)	18,221 (11.1)	27,533 (11.3)
70–74	12,044 (14.9)	13,770 (13.8)	2,208 (13.0)	2,561 (12.4)	13,470 (8.2)	20,506 (8.5)
75–79	5,813 (7.2)	6,563 (6.6)	1,375 (8.1)	1,575 (7.6)	8,830 (5.4)	13,465 (5.6)
Family history – no. (%)	2,774 (3.4)	4,745 (4.8)	550 (3.2)	939 (4.5)	9,908 (6.0)	16,898 (7.0)
Performance characteristics						
Intravenous sedation – no. (%)	72,466 (89.4)	95,416 (95.6)	15,409 (90.8)	19,879 (96.2)	152,186 (92.4)	234,587 (96.7)
Cecal intubation – no. (%)	79,803 (98.4)	97,978 (98.2)	16,567 (97.6)	20,138 (97.4)	160,600 (97.5)	235,565 (97.1)
Complications – no. (%)						
Bleeding	220 (0.27)	132 (0.13)	81 (0.48)	51 (0.25)	287 (0.17)	239 (0.10)
Perforation	22 (0.03)	24 (0.02)	8 (0.05)	5 (0.02)	23 (0.01)	52 (0.02)
Cardiorespiratory	30 (0.04)	36 (0.04)	9 (0.05)	16 (0.08)	45 (0.03)	65 (0.03)

Colonoscopy findings are presented in Table 2. 55.2% of screening, 54.9% of FOBT+, and 59.6% of diagnostic colonoscopies were performed in women. Screening colonoscopy revealed advanced adenoma in 5,295 women (5.3%) and in 7,923 men (9.8%) and cancer in 709 (0.7%) women and 1,061 (1.3%) men. FOBT+ colonoscopies identified advanced adenoma in 1,782 women (8.6%) and 2,522 men (14.9%) and cancer in 524 women (2.5%) and 746 men (4.4%). Upon diagnostic colonoscopy 6,977 women (2.9%) and 8,821 men (5.4%) were found to carry advanced adenoma, 1,802 women (0.7%) and 2,340 men (1.4%) had cancers. Remarkably, the highest rates of advanced adenoma and cancer were found in FOBT+ colonoscopies in both genders, reflecting the preselection of this screening group.

**Table 2:** Findings on 625,918 colonoscopies.

Age group - years	Gender	Screening colonoscopies		FOBT+ colonoscopies		Diagnostic colonoscopies	
		Cancerno. (%*)	Advanced adenomano. (%*)	Cancerno. (%*)	Advanced adenomano. (%*)	Cancerno. (%*)	Advanced adenomano. (%*)
18–39	women	-	-	3 (0.2)	28 (2.1)	73 (0.2)	295 (0.6)
	men	-	-	5 (0.5)	31 (3.2)	67 (0.2)	312 (1.0)
40–44	women	-	-	11 (0.9)	45 (3.5)	83 (0.3)	383 (1.5)
	men	-	-	17 (1.7)	60 (6.0)	79 (0.4)	488 (2.6)
45–49	women	-	-	24 (1.3)	113 (6.2)	116 (0.4)	648 (2.2)
	men	-	-	24 (1.3)	177 (9.4)	123 (0.6)	881 (4.2)
50–54	women	-	-	53 (1.5)	293 (8.1)	184 (0.6)	934 (2.9)
	men	-	-	62 (2.5)	350 (13.8)	207 (0.9)	1,284 (5.6)
55–59	women	114 (0.4)	1,306 (4.0)	58 (2.2)	215 (8.3)	187 (0.8)	758 (3.2)
	men	172 (0.7)	1,849 (7.8)	94 (4.6)	294 (14.5)	252 (1.6)	1,070 (6.9)
60–64	women	144 (0.6)	1,107 (4.9)	72 (2.9)	238 (9.7)	184 (0.9)	899 (4.2)
	men	173 (0.9)	1,737 (9.4)	93 (4.6)	345 (17.0)	294 (2.1)	1,083 (7.7)
65–69	women	206 (0.8)	1,441 (5.9)	105 (3.1)	344 (10.0)	329 (1.2)	1,231 (4.5)
	men	307 (1.5)	2,276 (10.9)	172 (5.9)	548 (18.6)	512 (2.8)	1,595 (8.8)
70–74	women	153 (1.1)	937 (6.8)	115 (4.5)	299 (11.7)	338 (1.7)	1,031 (5.0)
	men	255 (2.1)	1,359 (11.3)	153 (6.9)	413 (18.7)	437 (3.2)	1,241 (9.2)
75–79	women	92 (1.4)	504 (7.7)	83 (5.3)	207 (13.1)	308 (2.3)	798 (5.9)
	men	154 (2.6)	702 (12.1)	126 (9.2)	304 (22.1)	369 (4.19)	867 (9.8)

*Percentages are given related to the number of individuals in each group according to age, gender, and indication as presented in Table 1.

A logistic regression model was developed to predict the probabilities of advanced adenoma, carcinoma, and advanced neoplasia in all three colonoscopy groups according to gender. We found that the probability of finding advanced adenoma, cancer, or advanced neoplasia is doubled in men at any age in all three colonoscopy groups (Figure 1A–C). Furthermore, combined analysis of all colonoscopies revealed odds ratios for men compared to women for detection of advanced adenoma of 1.92 (95% CI: 1.87–1.96), for detection of cancer of 1.97 (95% CI: 1.88–2.07), and for detection of advanced neoplasia of 1.93 (95% CI: 1.89–1.97; Table 3, Table S2, and Figure S1).

**Table 3:** Predictors of advanced adenoma, carcinoma, and advanced neoplasia.

Parameter	Odds Ratio (OR)	95% CI of the OR	P
**Advanced Adenoma**			
Male gender	1.915	1.872–1.959	<.0001
Age (per year)	1.387	1.316–1.463	<.0001
Age^2^ (per year)	0.996	0.995–0.997	<.0001
Age^3^ (per year)	1.000	1.000–1.000	<.0001
FOBT+ vs. diagnostic colonoscopy	2.492	2.403–2.584	<.0001
Screening vs. diagnostic colonoscopy	1.246	1.215–1.278	<.0001
Colonoscopy complete vs. incomplete	1.201	1.112–1.296	<.0001
Sedation (yes vs. no)	0.991	0.948–1.036	0.7019
**Cancer**			
Male gender	1.971	1.878–2.068	<.0001
Age (per year)	0.977	0.897–1.064	0.5896
Age^2^ (per year)	1.002	1.000–1.003	0.0175
Age^3^ (per year)	1.000	1.000–1.000	0.0055
FOBT+ vs. diagnostic colonoscopy	2.701	2.529–2.885	<.0001
Screening vs. diagnostic colonoscopy	0.642	0.605–0.681	<.0001
Colonoscopy complete vs. incomplete	0.105	0.099–0.112	<.0001
Sedation (yes vs. no)	1.201	1.089–1.324	0.0002
**Advanced Neoplasia**			
Male gender	1.933	1.893–1.974	<.0001
Age (per year)	1.297	1.238–1.358	<.0001
Age^2^ (per year)	0.997	0.996–0.998	<.0001
Age^3^ (per year)	1.000	1.000–1.000	<.0001
FOBT+ vs. diagnostic colonoscopy	2.614	2.529–2.702	<.0001
Screening vs. diagnostic colonoscopy	1.133	1.107–1.161	<.0001
Colonoscopy complete vs. incomplete	0.443	0.421–0.465	<.0001
Sedation (yes vs. no)	1.026	0.984–1.069	0.2324

Advanced neoplasia includes advanced adenoma and cancer and has been suggested as the most appropriate target for colorectal cancer screening [4], [14], [16], [18], [23], [25]–[27]. To determine whether screening for advanced neoplasia should start earlier in men than in women prevalence data of advanced adenoma from age groups below the current start age of screening are required. Our analysis reveals that the number of colonoscopies needed to detect cancer or advanced adenoma is higher in women than in men at all ages and in all three colonoscopy indications analyzed (Table 4). At age 75 a total of 14.8 (95% CI, 14.4–15.2) screening, 18.2 (95% CI, 17.7–18.7) diagnostic, or 7.9 (95% CI, 7.6–8.2) FOBT+ colonoscopies were needed to find advanced adenoma in women. Comparable numbers were reached up to twenty years earlier in men. At age 50, the age screening is usually initiated, 39.0 (95% CI, 38.0–40.0) diagnostic, and 16.3 (95% CI, 15.7–16.9) FOBT+ colonoscopies were required to find advanced adenoma in women. Similar numbers were reached in men between age 40 to 41 with 41.5 (95% CI, 40.0–43.0) to 38.1 (95% CI, 36.8–39.4) diagnostic colonoscopies, and 17.2 (16.5–18.1) to 15.9 (15.2–16.6) FOBT+ colonoscopies, respectively.

**Table 4:** Number of colonoscopies needed to identify cancer or advanced adenoma.

Indication	Age(yrs)	Cancer		Advanced adenoma	
		Malen (95% CI)	Femalen (95% CI)	Malen (95% CI)	Femalen (95% CI)
**Screening Colonoscopies**					
	75	51.8 (48.7–55.0)	101.0 (94.6–107.9)	8.2 (8.0–8.4)	14.8 (14.4–15.2)
	70	63.8 (60.6–67.3)	124.9 (117.9–132.3)	9.0 (8.8–9.1)	16.2 (15.9–16.6)
	65	82.4 (78.2–87.0)	161.5 (152.4–171.2)	9.9 (9.7–10.1)	18.1 (17.7–18.5)
	60	110.2 (104.3–116.5)	216.3 (203.8–229.6)	11.3 (11.1–11.6)	20.8 (20.3–21.2)
	55	151.2 (142.2–160.7)	297.0 (278.2–317.0)	13.5 (13.2–13.8)	24.9 (24.2–25.5)
**FOBT+ Colonoscopies**					
	75	13.1 (12.3–13.9)	24.8 (23.1–26.6)	4.6 (4.5–4.7)	7.9 (7.6–8.2)
	70	15.9 (15.0–16.9)	30.5 (28.5–32.5)	5.0 (4.8–5.1)	8.6 (8.3–8.9)
	65	20.4 (19.1–21.7)	39.2 (36.6–419)	5.5 (5.3–5.6)	9.5 (9.2–9.9)
	60	27.0 (25.3–28.7)	52.2 (48.8–55.8)	6.2 (6.0–6.3)	10.9 (10.5–11.3)
	55	36.7 (34.3–39.2)	71.4 (66.5–76.6)	7.2 (7.0–7.5)	12.9 (12.5–13.4)
	50	50.8 (47.1–54.7)	99.1 (91.7–107.2)	9.0 (8.7–9.3)	16.3 (15.7–16.9)
	45	70.8 (65.0–77.1)	138.6 (126.8–151.4)	11.9 (11.5–12.4)	21.9 (21.0–22.8)
	40	98.3 (89.2–108.4)	192.8 (174.4–213.1)	17.2 (16.5–18.1)	32.1 (30.6–33.7)
**Diagnostic Colonoscopies**					
	75	33.6 (31.9–35.3)	65.2 (61.8–68.8)	10.0 (9.7–10.2)	18.2 (17.7–18.7)
	70	41.4 (39.5–43.3)	80.5 (76.7–84.6)	10.9 (10.7–11.2)	20.0 (19.5–20.4)
	65	53.3 (50.8–55.9)	104.0 (98.9–109.4)	12.1 (11.8–12.4)	22.3 (21.8–22.8)
	60	71.1 (67.9–74.5)	139.2 (132.5–146.3)	13.9 (13.6–14.2)	25.6 (25.0–26.2)
	55	97.4 (92.8–102.3)	191.0 (181.4–201.1)	16.5 (16.2–16.9)	30.7 (30.0–31.5)
	50	135.5 (128.0–143.4)	266.1 (250.7–282.4)	20.8 (20.3–21.4)	39.0 (38.0–40.0)
	45	189.5 (177.0–202.1)	372.5 (347.1–399.7)	28.2 (27.4–29.0)	53.1 (51.5–54.7)
	40	263.8 (243.2–286.1)	518.9 (477.6–563.8)	41.5 (40.0–43.0)	78.5 (75.6–81.5)

## Discussion

The lifetime risk of colorectal cancer is similar in women and in men [28], but screening studies including individuals 50 years of age and above have demonstrated that advanced adenoma and cancer occur at an earlier age in men [14], [17], [18]. Still, more detailed information on the risk of advanced colonic neoplasia in women and men below the age of 50 is needed for the implementation of modifications of screening guidelines concerning the age of onset of screening dependent on gender [20]. In our study of 625,918 persons who received colonoscopies in Bavaria between 2006 and 2008, the large number of participants allowed us to derive a reliable statistical model to predict the age-dependent risks of women and men for advanced adenoma and cancer. We found that throughout all age groups the risk of advanced neoplasia was higher in men than in women and nearly twice as many cases of advanced colorectal neoplasia were detected in men than in women of same age.

Since colorectal cancer rates begin to increase in the sixth decade of life in average-risk individuals, an age of 50 years has been arbitrarily selected as an appropriate age to initiate colonoscopy screening for both genders [9], [10], [12]. However, age-adjusted incidence and mortality rates of colorectal cancer are higher in men than in women who reach the levels observed in men 4 to 10 years later [19]. Similarly, screening studies have demonstrated that during their sixth decade women have lower absolute risks of advanced neoplasia than men [16], [18]. We demonstrate that men reach the same risk of advanced neoplasia 10 and more years earlier than women. Our data are in line with findings of screening studies [20] but by including diagnostic and FOBT+ colonoscopies allow for the first time more precise risk estimations for neoplastic and preneoplastic lesions in women and men before the age of 50 in a general risk population. Moreover, in contrast to screening studies, which only include samples of volunteers [4], [14], [16], [18], [23], [25], [26], [29] our data are based on a near-complete census of all outpatient colonoscopies in the Bavarian population. These data allow estimating the risk of the general population below the age of 50 while previous studies have only included few individuals of this age group [29] or included younger individuals at increased risk only [16]. As demonstrated by our analysis, also non-screening colonoscopies performed in a given population can be used to identify the age at which men and women reach comparable risks. The age differences in numbers of colonoscopies needed to detect advanced lesions are similar in all three colonoscopy groups analyzed. In case of a negative screening colonoscopy re-colonoscopy is commonly recommended after 10 years [9], [10]. Due to the low risk of colorectal cancer and advanced adenomas 10 and more years after negative colonoscopy an extension of this interval has been suggested [7]. Yet, it remains to be studied, whether gender-dependent differences of risk need to be taken into account when discussing the extension of screening intervals.

The performance rate as determined by the cecum rate was high in our setting and comparable to other reports or higher and within the range expected for expert colonoscopists [16], [23], [30]. The data presented here document that colonoscopy is safe in both the screening and diagnostic settings. The rate of advanced neoplasia found in the screening and diagnostic settings is within the range of other studies reporting frequencies of advanced neoplasia between 5.6 and 10.6% for both genders [4], [14], [16], [18], [25]. The higher prevalence of cancer in the diagnostic and FOBT+ groups is explained by the fact that these colonoscopies were performed because of complaints or occult blood in stool. In contrast, the higher prevalence of advanced adenoma in both screening populations suggests that people at higher risk of colorectal cancer including those with a specific family history might more frequently accept the offer of screening [13]. Moreover, the higher probability of asymptomatic individuals to be diagnosed with advanced adenoma or advanced neoplasia upon colonoscopy than symptomatic individuals might also be influenced by the fact that symptomatic individuals receive colonoscopies more frequently in a hospital setting. The decline in the number of diagnostic colonoscopies at the age of 55 when screening colonoscopy is offered might indicate that a certain number of diagnostic colonoscopies before the age of 55 may have been performed for subtle symptoms or for screening but were not documented as screening colonoscopies before the age of 55.

Similar to previous reports, we also observed that women outnumber men in screening colonoscopies [16]. Interestingly, this also holds true for the other colonoscopy indications suggesting a greater health consciousness of women [26]. It has been discussed that more men than women receive a screening colonoscopy for subtle symptoms potentially related to colorectal cancer and that this selection bias might be the reason for the higher prevalence of advanced neoplasia in men [16]. However, our data demonstrate that male gender is associated with a higher probability of advanced neoplasia independent of the indication for colonoscopy. Sex-related biologic differences like hormonal effects before menopause and hormone-replacement therapy may in addition to different life styles exert a protective effect and explain the reduced risk for colorectal cancer in women [31]. Still, participation in screening colonoscopy in Bavaria remains low in both women and men. However, the participation rate in other population-level colorectal cancer screening programs throughout the world is currently unknown [1]. Similar as in the case of screening colonoscopy, women also more frequently accept the offer for FOBT screening in Bavaria. Our data confirm the well-known fact that pre-screening by FOBT results in a lower number of colonoscopies needed to identify cancer or advanced adenoma. However, its overall sensitivity for advanced colonic lesions is very limited compared to colonoscopy [4], [5], [32].

We demonstrate that levels of incidence of advanced neoplasia differ strongly between both genders depending on age. This suggests that women do not benefit from a start of screening at age 50 or 55 years to the same extent as men. Currently, either women might be subjected to screening unnecessarily early in life or start of screening might be delayed in men. It has been suggested that compliance with screening recommendations may improve if individuals understand that recommendations for screening are based on their personal risk, rather than the selection of an arbitrary age [33]. The increased risk of family history has been widely acknowledged by screening guidelines [9], [10], [12]. However, a gender-specific recommendation of age for start of screening for colorectal cancer might even be more relevant from a public health point of view than the differentiation by family history [13]. The American College of Gastroenterology suggests starting screening in male African Americans at age 45 in contrast to women and white men [12]. Yet, the evidence for this recommendation is mainly based on cancer epidemiology data [11]. Next to gender, family history, and race, other factors such as smoking status, alcohol consumption, obesity, and diabetes mellitus [32] might also be taken into account in the future to define the individual risk. Still, it needs to be kept in mind that more complicated recommendations and the choice between various screening modalities might also result in confusion and non-compliance with screening guidelines [34].

A limitation of this study is that family history was documented less detailed than in some screening studies [16]. Since our data picture the common practice in everyday outpatient care, and taking detailed family histories may not regularly be part of this routine, our data may give a valid impression of the population mix of individuals at general risk and persons from family risk populations. Moreover, assuming a similar distribution of family risk between men and women, inclusion of family history in our model would not result in a major change in the gender-dependent differences reported. Three factors may have influenced the number of advanced neoplasia included in the analysis: Colonoscopies done in privately insured patients and colonoscopies performed in hospitals are not documented in the data base. As the majority of the inhabitants of Bavaria are members of the CHI system and colonoscopies performed in hospitals are generally done in more severely ill patients, this might be a negligible bias or, at best, result in an underestimate of advanced neoplasia. Only complete documentations on size and histology resulted in inclusion of lesions into the analysis, this also might have resulted in an underestimate. Similar as in other large studies [16] no central pathological review of lesions has been performed and it is unknown whether the potential misclassification of lesions has a relevant impact on our database. However, a potential bias due to these three factors would most likely only affect the total number of lesions but not the relative distribution of lesions between men and women and, therefore, not affect the gender-dependent differences either.

In summary, male gender is a risk factor for finding advanced adenoma or cancer upon colonoscopy independent of the indication for colonoscopy. At any age between 40 and 79 years, men are at higher risk of having advanced neoplasia diagnosed upon colonoscopy than women. This suggests that starting screening earlier in life in men than in women might result in a relevant increase in the detection of asymptomatic preneoplastic and neoplastic colonic lesions.

## Supporting Information

Methods S1 (PDF)

Table S1 **Indications for diagnostic colonoscopies.**
(PDF)

Table S2 **Regression coefficients.**
(PDF)

Figure S1 **Relative risk (RR) of advanced neoplasia in men compared to women depending on age.** The black line shows the observed, unadjusted RR at each year of age. The band marks the corresponding 95% confidence intervals.
(PDF)
