# Supplementary material for: Risk of Advanced Colorectal Neoplasia According to Age and Gender
Source: PLoS One. 2011 May 24;6(5):e20076. doi: 10.1371/journal.pone.0020076 (PMC3101231; doi:10.1371/journal.pone.0020076)
Supplement: Methods S1 — (PDF) [file pone.0020076.s001.pdf]

Supplementary methods S1

The linear predictor LP is the sum of the intercept and the individual values of the independent variables weighted by the corresponding regression coefficients b that can be derived from Supplementary Table 2:

$$LP = intercept + b_{male} \times male + b_{age} \times age + b_{age\ squared} \times age^2 + b_{age\ cubed} \times age^3$$
$$+ b_{screening} \times screening + b_{FOBT} \times FOBT + b_{complete} \times complete + b_{sedation} \times sedation$$

where:

- Male = 1 if the patient is male and male = 0 otherwise,
- Age, age^2, and age^3 are the numeric values in years,
- Screening = 1 in case of a screening colonoscopy and screening = 0 otherwise,
- FOBT = 1 in case of a positive FOBT and FOBT = 0 otherwise,
- Complete = 1 if the colonoscopy was complete and complete = 0 otherwise,
- Sedation = 1 if the colonoscopy was performed under intravenous sedation, and sedation = 0 otherwise.

The linear predictor can be used to calculate the outcome probability P:

$$P = 1 / [\exp(-LP) + 1].$$

Since the number of colonoscopies needed to detect a given lesion was defined as the reciprocal 1/P of the probability, its point estimate can be given as:

$$\text{Number of colonoscopies} = \exp(-LP) + 1.$$

For example, the linear predictor for advanced neoplasia in a man aged 50 who receives a complete diagnostic colonoscopy to investigate the reason of a positive FOBT under sedation is:

$$LP = -9.9206 + 0.6590 \times 1 + 0.2598 \times 50 - 0.00291 \times 50^2 + 0.000012 \times 50^3 + 0.9608 \times 1 + 0.1253 \times 0$$
$$- 0.8151 \times 1 + 0.0253 \times 1 =$$
$$-1.8756$$

This translates to a predicted probability of 13.3% and number of colonoscopies of 7.5.
