# Supplementary material for: Risk of Advanced Colorectal Neoplasia According to Age and Gender
Source: PLoS One. 2011 May 24;6(5):e20076. doi: 10.1371/journal.pone.0020076 (PMC3101231; doi:10.1371/journal.pone.0020076)
Supplement: Table S1 — Indications for diagnostic colonoscopies. (PDF) [file pone.0020076.s002.pdf]

Table S1: Indications for diagnostic colonoscopies.

**Indication for colonoscopy**	**Women N (%)**	**Men N (%)**	**All N (%)**
Overt blood	40,297 (15.3)	36,252 (20)	76,549 (17.2)
Diarrhoea	40,735 (15.5)	26,776 (14.7)	67,511 (15.2)
Abdominal pain	123,022 (46.7)	71,031 (39.1)	194,053 (43.6)
Change in bowel habits	34,534 (13.1)	22,212 (12.2)	56,746 (12.8)
Anemia	10,686 (4.1)	6,546 (3.6)	17,232 (3.9)
Weight loss	7,581 (2.9)	5,819 (3.2)	13,400 (3.0)
constipation	20,260 (7.7)	7,585 (4.2)	27,845 (6.3)
Pain with defecation	3,022 (1.1)	2,157 (1.2)	5,179 (1,2)
Perianal pain	3,716 (1.4)	3,142 (1.7)	6,858 (1,5)
Incomplet emptying of bowel	1,439 (0.5)	743 (0.4)	2,182 (0.5)
Incontinency	1,141 (0.4)	464 (0.3)	1,605 (0.4)
Prurits ani	1,517 (0.6)	1,496 (0.8)	3,013 (0.7)
Other	51,643 (19.6)	38,599 (21.3)	90,242 (20.3)

More than one check was allowed, therefore, percentages do not sum up to 100.
