# Supplementary material for: Risk of Advanced Colorectal Neoplasia According to Age and Gender
Source: PLoS One. 2011 May 24;6(5):e20076. doi: 10.1371/journal.pone.0020076 (PMC3101231; doi:10.1371/journal.pone.0020076)
Supplement: Table S2 — Regression coefficients. (PDF) [file pone.0020076.s003.pdf]

Table S2: Regression coefficients

Parameter	Regression Coefficient	Standard Error	Wald χ^2 (df = 1)
**Advanced Adenoma**			
Intercept	-12.2826	0.4895	629.6160
Male gender	0.6499	0.0116	3148.5532
Age (per year)	0.3273	0.0270	147.2955
Age^2 (per year)	-0.0040	4.87E-4	68.7714
Age^3 (per year)	1.8E-5	2.87E-6	37.8024
FOBT+ vs. diagnostic colonoscopy	0.9130	0.0185	2448.8233
Screening vs. diagnostic colonoscopy	0.2201	0.0130	285.0738
Colonoscopy complete vs. incomplete	0.1829	0.0391	21.8285
Sedation (yes vs. no)	-0.00866	0.0226	0.1465
**Cancer**			
Intercept	-5.4444	0.7612	51.1518
Male gender	0.6785	0.0246	761.9523
Age (per year)	-0.0234	0.0434	0.2909
Age^2 (per year)	0.00191	0.0008	5.6477
Age^3 (per year)	0.00001	4.813E-6	7.6921
FOBT+ vs. diagnostic colonoscopy	0.9935	0.0336	874.0207
Screening vs. diagnostic colonoscopy	-0.4433	0.0298	221.1735
Colonoscopy complete vs. incomplete	-2.2523	0.0322	4890.9582
Sedation (yes vs. no)	0.1828	0.0497	13.5042
**Advanced Neoplasia**			
Intercept	-9.9206	0.4229	550.3677
Male gender	0.6590	0.0107	3763.4336
Age (per year)	0.2598	0.0235	122.2371
Age^2 (per year)	-0.00291	0.000427	46.5506
Age^3 (per year)	0.000012	2.525E-6	22.0707
FOBT+ vs. diagnostic colonoscopy	0.9608	0.0169	3233.2729
Screening vs. diagnostic colonoscopy	0.1253	0.0121	106.3655
Colonoscopy complete vs. incomplete	-0.8151	0.0253	1036.0925
Sedation (yes vs. no)	0.0253	0.0212	1.4261
