# Supplementary material for: Risk of Advanced Colorectal Neoplasia According to Age and Gender
Source: PLoS One. 2011 May 24;6(5):e20076. doi: 10.1371/journal.pone.0020076 (PMC3101231; doi:10.1371/journal.pone.0020076)

Figure S1: Relative risk (RR) of advanced neoplasia in men compared to women depending on age. The black line shows the observed, unadjusted RR at each year of age. The band marks the corresponding 95% confidence intervals.

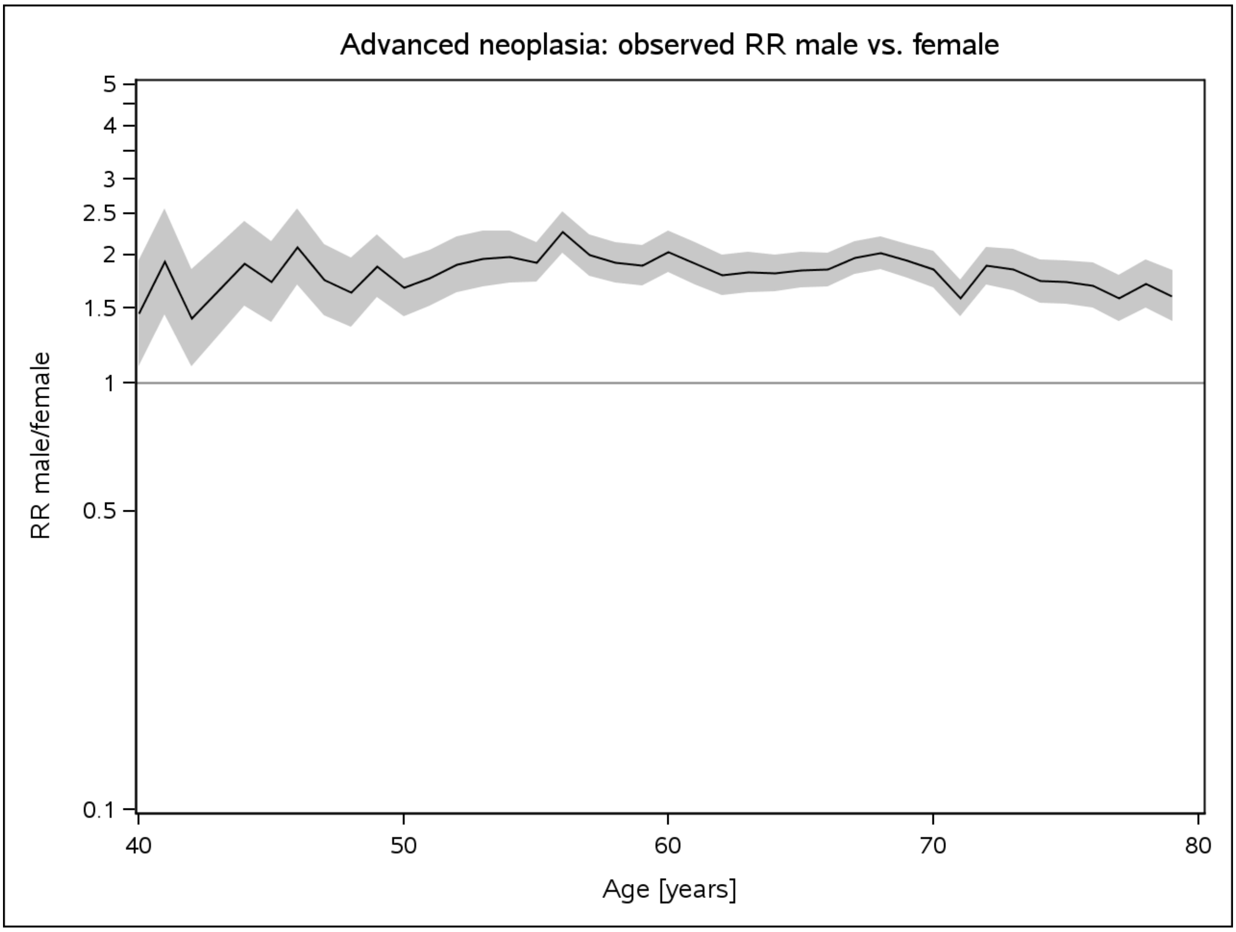

Supplement: Figure S1 — Relative risk (RR) of advanced neoplasia in men compared to women depending on age. The black line shows the observed, unadjusted RR at each year of age. The band marks the corresponding 95% confidence intervals. (PDF) [file pone.0020076.s004.pdf]
